# 5-Diethyl­amino-2-{(*E*)-[(3-iodo­phen­yl)imino]­meth­yl}phenol

**DOI:** 10.1107/S1600536812022556

**Published:** 2012-05-31

**Authors:** Hilal Vesek, Canan Kazak, Erbil Ağar, Sümeyye Gümüş

**Affiliations:** a Department of Physics, Faculty of Arts and Sciences, Ondokuz Mayıs University, Kurupelit, TR-55139 Samsun, Turkey; b Department of Chemistry, Faculty of Arts and Sciences, Ondokuz Mayıs University, Kurupelit, TR-55139 Samsun, Turkey

## Abstract

The title Schiff base, C_17_H_19_IN_2_O, is not planar, displaying a dihedral angle of 34.9 (2)° between the two aromatic rings. The mol­ecular conformation allows the formation of a strong intra­molecular O—H⋯N hydrogen bond with graph-set motif *S*(6) between the hy­droxy group and the imine N atom.

## Related literature

For Schiff base tautomerism, see: Cohen *et al.* (1964 ▶); Hadjoudis *et al.* (1987 ▶). For the biological properties of Schiff bases, see: Dao *et al.* (2000 ▶). For related structures, see: Gül, Ağar & Işık (2007 ▶); Gül, Erşahin, Ağar & Işık (2007 ▶); Pekdemir *et al.* (2012 ▶); Yüce *et al.* (2004 ▶); Demirtaş *et al.* (2011 ▶). For the classification of hydrogen-bonding patterns, see: Bernstein *et al.* (1995 ▶).
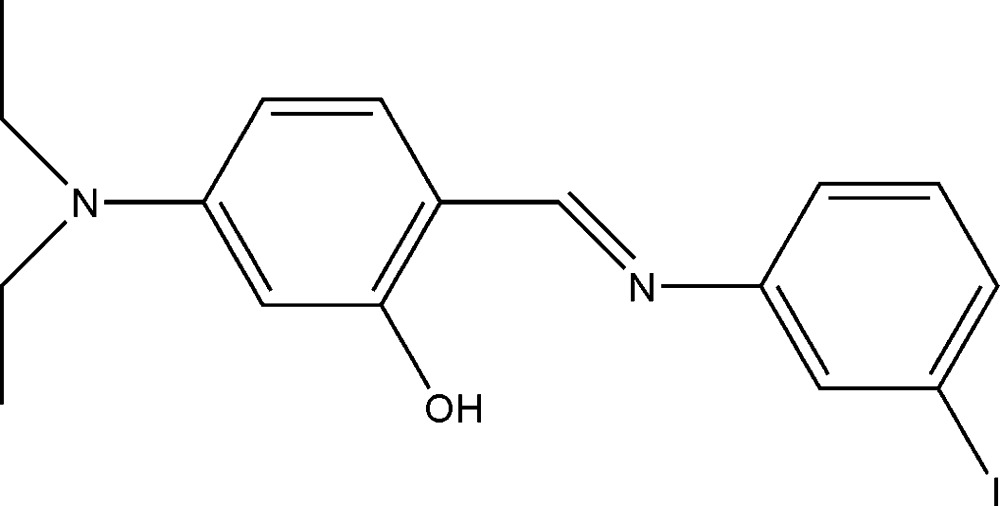


## Experimental

### 

#### Crystal data

C_17_H_19_IN_2_O
*M* *_r_* = 394.24
Orthorhombic, 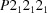

*a* = 6.6999 (6) Å
*b* = 15.248 (2) Å
*c* = 16.1195 (15) Å
*V* = 1646.7 (3) Å^3^
*Z* = 4
Mo *K*α radiation
μ = 1.95 mm^−1^
*T* = 296 K
0.49 × 0.34 × 0.21 mm

#### Data collection

Stoe IPDS II diffractometer
Absorption correction: integration (*X-RED32*; Stoe & Cie, 2002 ▶) *T*_min_ = 0.451, *T*_max_ = 0.603
6072 measured reflections
3225 independent reflections
2417 reflections with *I* > 2σ(*I*)
*R*_int_ = 0.049

#### Refinement

*R*[*F* ^2^ > 2σ(*F* ^2^)] = 0.037
*wR*(*F* ^2^) = 0.074
*S* = 0.86
3225 reflections
190 parameters
H-atom parameters constrained
Δρ_max_ = 0.57 e Å^−3^
Δρ_min_ = −0.63 e Å^−3^
Absolute structure: Flack (1983 ▶), 1355 Friedel pairs
Flack parameter: −0.02 (3)

### 

Data collection: *X-AREA* (Stoe & Cie, 2002 ▶); cell refinement: *X-AREA*; data reduction: *X-RED32* (Stoe & Cie, 2002 ▶); program(s) used to solve structure: *SHELXS97* (Sheldrick, 2008 ▶); program(s) used to refine structure: *SHELXL97* (Sheldrick, 2008 ▶); molecular graphics: *ORTEP-3 for Windows* (Farrugia, 1997 ▶); software used to prepare material for publication: *WinGX* (Spek, 2009 ▶).

## Supplementary Material

Crystal structure: contains datablock(s) I, global. DOI: 10.1107/S1600536812022556/bh2429sup1.cif

Structure factors: contains datablock(s) I. DOI: 10.1107/S1600536812022556/bh2429Isup2.hkl

Supplementary material file. DOI: 10.1107/S1600536812022556/bh2429Isup3.cml

Additional supplementary materials:  crystallographic information; 3D view; checkCIF report

## Figures and Tables

**Table 1:** Hydrogen-bond geometry (Å, °)

*D*—H⋯*A*	*D*—H	H⋯*A*	*D*⋯*A*	*D*—H⋯*A*
O1—H1⋯N1	0.82	1.85	2.577 (6)	147

**Fig. 1.**
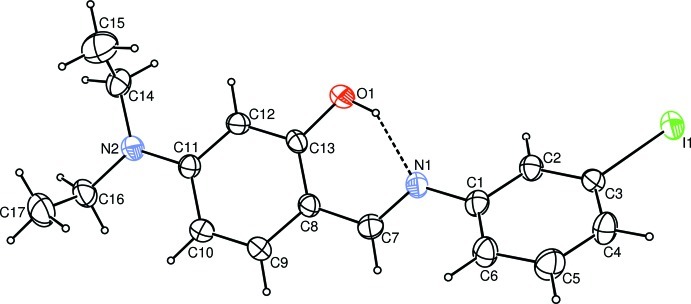
The molecular structure of the title compound, showing displacement ellipsoids at the 30% probability level.

## supplementary crystallographic information

### Comment

Schiff base compounds are used in many different areas. Generally, they exhibit biological activity: anti-bacterial and anti-cancer properties were demonstrated (Dao *et al.*, 2000). Thermochromic and photochromic Schiff base compounds can be classified on the basis of their specific characteristics (Cohen *et al.*, 1964): intermolecular hydrogen bonds may be formed in two different ways. Chromic action is strongly related to the tautomerization between the O—H···N═C—C═C (enolimino) and —C═ O···H—N—C═C—C (ketoamino) tautomeric forms (Hadjoudis *et al.*, 1987). The title compound is stabilized in the phenol-imine tautomeric form (Fig. 1).

The C1—N1 bond length, 1.407 (7) Å, is in agreement with the distance reported for 2-[(*E*)-(naphthalen-2-ylimino)methyl]-4-(trifluoromethoxy)phenol [1.417 (2) Å, Pekdemir *et al.*, 2012] and 1-{4-[(2-hydroxybenzylidene)amino]phenyl}ethanone [1.4138 (17) Å, Yüce *et al.*, 2004]. The C7═N1 bond length of 1.292 (7) Å is also comparable to the imine double bond found in (*E*)-4-bromo-2-[(2-hydroxy-5-methylphenyl)iminomethy]phenol [1.289 (6) Å, Gül, Ağar & Işık, 2007]. Figure 1 also shows a strong intramolecular hydrogen bond O1—H1···N1, which can be described as an *S*(6) motif (Bernstein *et al.*, 1995). The O1···N1 separation of 2.577 (6) Å is comparable to that observed for similar hydrogen bonds in related Schiff bases (Gül, Erşahin, Ağar & Işık, 2007). The C3—I1 bond length, 2.114 (5) Å, is in agreement with other C—I bonds, for example in 2-(2-iodophenyl)isoindoline-1,3-dione [2.094 (3) Å; Demirtaş *et al.*, 2011]. The title molecule is not planar, displaying a dihedral angle of 34.9 (2)° between the two aromatic rings.

### Experimental

The title compound was prepared by refluxing for 1 h. a mixture of 4-(diethylamino)-2-hydroxybenzaldehyde (0.022 g, 0.11 mmol) in ethanol (20 ml) and 3-iodoaniline (0.025 g, 0.11 mmol) in ethanol (20 ml). Crystals suitable for X-ray analysis were obtained from ethanol by slow evaporation (yield: 72%, m.p. 394–395 K).

### Refinement

The H atom of the hydroxy group was refined with the O1—H1 bond length constrained to 0.82 Å and *U*_iso_(H1) = 1.5*U*_eq_(O1). All other H atoms were placed in calculated positions and constrained to ride on their parents atoms, with C—H = 0.93–0.97 Å and *U*_iso_(H) = 1.2*U*_eq_(C) or 1.5*U*_eq_(C). The refinement of the Flack parameter (Flack, 1983) is based on 1355 measured Friedel pairs.

### Crystal data

**Table:**

C_17_H_19_IN_2_O	*D*_x_ = 1.590 Mg m^−^^3^
*M**_r_* = 394.24	Melting point: 394 K
Orthorhombic, *P*2_1_2_1_2_1_	Mo *K*α radiation, λ = 0.71073 Å
Hall symbol: P 2ac 2ab	Cell parameters from 8061 reflections
*a* = 6.6999 (6) Å	θ = 1.8–28.1°
*b* = 15.248 (2) Å	µ = 1.95 mm^−^^1^
*c* = 16.1195 (15) Å	*T* = 296 K
*V* = 1646.7 (3) Å^3^	Prism, yellow
*Z* = 4	0.49 × 0.34 × 0.21 mm
*F*(000) = 784	

### Data collection

**Table:**

Stoe IPDS II diffractometer	3225 independent reflections
Radiation source: fine-focus sealed tube	2417 reflections with *I* > 2σ(*I*)
Graphite monochromator	*R*_int_ = 0.049
Detector resolution: 6.67 pixels mm^-1^	θ_max_ = 26.0°, θ_min_ = 1.8°
rotation method scans	*h* = −6→8
Absorption correction: integration (*X-RED32*; Stoe & Cie, 2002)	*k* = −15→19
*T*_min_ = 0.451, *T*_max_ = 0.603	*l* = −20→20
6072 measured reflections	

### Refinement

**Table:**

Refinement on *F*^2^	Secondary atom site location: difference Fourier map
Least-squares matrix: full	Hydrogen site location: inferred from neighbouring sites
*R*[*F*^2^ > 2σ(*F*^2^)] = 0.037	H-atom parameters constrained
*wR*(*F*^2^) = 0.074	*w* = 1/[σ^2^(*F*_o_^2^) + (0.0337*P*)^2^] where *P* = (*F*_o_^2^ + 2*F*_c_^2^)/3
*S* = 0.86	(Δ/σ)_max_ = 0.002
3225 reflections	Δρ_max_ = 0.57 e Å^−^^3^
190 parameters	Δρ_min_ = −0.63 e Å^−^^3^
0 restraints	Absolute structure: Flack (1983), 1355 Friedel pairs
0 constraints	Flack parameter: −0.02 (3)
Primary atom site location: structure-invariant direct methods	

### Fractional atomic coordinates and isotropic or equivalent isotropic displacement parameters (Å^2^)

**Table:**

	*x*	*y*	*z*	*U*_iso_*/*U*_eq_	
C1	0.5355 (9)	0.7994 (3)	0.3130 (3)	0.0424 (14)	
C2	0.6547 (9)	0.8639 (3)	0.2779 (3)	0.0438 (13)	
H2	0.7869	0.8699	0.2946	0.053*	
C3	0.5784 (8)	0.9186 (3)	0.2188 (3)	0.0411 (13)	
C4	0.3846 (10)	0.9108 (4)	0.1905 (4)	0.0539 (16)	
H4	0.3353	0.9477	0.1494	0.065*	
C5	0.2675 (12)	0.8468 (4)	0.2254 (4)	0.0648 (16)	
H5	0.1361	0.8409	0.2077	0.078*	
C6	0.3395 (9)	0.7908 (4)	0.2860 (4)	0.0513 (15)	
H6	0.2573	0.7478	0.3085	0.062*	
C7	0.5263 (10)	0.7053 (4)	0.4284 (3)	0.0463 (16)	
H7	0.3934	0.7205	0.4366	0.056*	
C8	0.6183 (8)	0.6397 (4)	0.4808 (4)	0.0398 (14)	
C9	0.5079 (9)	0.5976 (4)	0.5432 (4)	0.0470 (14)	
H9	0.3765	0.6148	0.5522	0.056*	
C10	0.5856 (9)	0.5330 (4)	0.5909 (3)	0.0474 (15)	
H10	0.5071	0.5067	0.6314	0.057*	
C11	0.7872 (8)	0.5048 (3)	0.5794 (3)	0.0396 (12)	
C12	0.8970 (8)	0.5476 (4)	0.5182 (3)	0.0429 (13)	
H12	1.0285	0.5305	0.5091	0.052*	
C13	0.8187 (8)	0.6140 (4)	0.4706 (3)	0.0382 (14)	
C14	1.0628 (11)	0.4029 (4)	0.6072 (4)	0.0602 (17)	
H14A	1.1138	0.3722	0.6555	0.072*	
H14B	1.1530	0.4512	0.5956	0.072*	
C15	1.0651 (14)	0.3412 (6)	0.5345 (5)	0.098 (3)	
H15A	1.1984	0.3200	0.5259	0.147*	
H15B	1.0204	0.3715	0.4858	0.147*	
H15C	0.9779	0.2926	0.5455	0.147*	
C16	0.7536 (12)	0.3977 (4)	0.6930 (3)	0.0594 (15)	
H16A	0.6713	0.4420	0.7194	0.071*	
H16B	0.8452	0.3752	0.7345	0.071*	
C17	0.6208 (11)	0.3235 (5)	0.6640 (6)	0.085 (2)	
H17A	0.5497	0.2997	0.7105	0.127*	
H17B	0.7012	0.2785	0.6392	0.127*	
H17C	0.5273	0.3454	0.6239	0.127*	
I1	0.76828 (6)	1.01529 (2)	0.16772 (2)	0.05438 (13)	
N1	0.6279 (7)	0.7428 (3)	0.3703 (3)	0.0431 (11)	
N2	0.8680 (7)	0.4386 (3)	0.6273 (3)	0.0478 (12)	
O1	0.9360 (6)	0.6522 (3)	0.4129 (2)	0.0532 (11)	
H1	0.8727	0.6905	0.3888	0.080*	

### Atomic displacement parameters (Å^2^)

**Table:**

	*U*^11^	*U*^22^	*U*^33^	*U*^12^	*U*^13^	*U*^23^
C1	0.056 (3)	0.032 (3)	0.039 (3)	0.004 (3)	0.001 (2)	−0.006 (2)
C2	0.048 (3)	0.044 (3)	0.039 (3)	0.000 (3)	0.000 (2)	0.002 (2)
C3	0.042 (3)	0.037 (3)	0.044 (3)	0.002 (2)	0.011 (2)	0.004 (2)
C4	0.061 (4)	0.049 (4)	0.051 (4)	0.000 (3)	−0.017 (3)	0.008 (3)
C5	0.049 (4)	0.062 (3)	0.084 (4)	−0.002 (4)	−0.015 (4)	0.008 (3)
C6	0.051 (4)	0.041 (3)	0.061 (4)	−0.006 (3)	−0.008 (3)	0.005 (3)
C7	0.055 (4)	0.043 (4)	0.040 (3)	0.008 (3)	0.006 (3)	−0.008 (3)
C8	0.047 (3)	0.032 (3)	0.040 (3)	−0.003 (3)	0.007 (3)	−0.001 (3)
C9	0.043 (3)	0.047 (4)	0.051 (4)	−0.003 (3)	0.011 (3)	0.003 (3)
C10	0.046 (3)	0.051 (4)	0.045 (3)	0.000 (3)	0.009 (2)	0.004 (3)
C11	0.041 (3)	0.036 (3)	0.041 (2)	0.000 (3)	−0.002 (2)	−0.0006 (17)
C12	0.040 (3)	0.042 (3)	0.046 (3)	0.001 (2)	0.008 (3)	−0.002 (3)
C13	0.046 (4)	0.032 (3)	0.037 (3)	−0.006 (2)	0.007 (2)	−0.002 (2)
C14	0.063 (4)	0.056 (4)	0.061 (4)	0.010 (3)	−0.007 (3)	0.013 (3)
C15	0.108 (7)	0.089 (6)	0.098 (7)	0.025 (5)	0.021 (5)	−0.012 (5)
C16	0.064 (4)	0.061 (3)	0.054 (3)	−0.001 (5)	0.002 (4)	0.018 (2)
C17	0.072 (5)	0.069 (5)	0.114 (6)	−0.019 (4)	0.003 (5)	0.024 (5)
I1	0.0585 (2)	0.05387 (18)	0.05074 (17)	−0.0006 (2)	0.0024 (2)	0.01511 (17)
N1	0.054 (3)	0.036 (2)	0.039 (2)	−0.001 (2)	0.004 (2)	0.003 (2)
N2	0.050 (3)	0.052 (3)	0.041 (2)	0.003 (2)	0.005 (2)	0.011 (2)
O1	0.051 (3)	0.060 (3)	0.048 (3)	0.008 (2)	0.015 (2)	0.013 (2)

### Geometric parameters (Å, º)

**Table:**

C1—C2	1.388 (7)	C11—N2	1.381 (7)
C1—C6	1.390 (8)	C11—C12	1.393 (7)
C1—N1	1.407 (7)	C12—C13	1.374 (7)
C2—C3	1.365 (7)	C12—H12	0.9300
C2—H2	0.9300	C13—O1	1.349 (6)
C3—C4	1.381 (8)	C14—N2	1.450 (8)
C3—I1	2.114 (5)	C14—C15	1.503 (10)
C4—C5	1.373 (8)	C14—H14A	0.9700
C4—H4	0.9300	C14—H14B	0.9700
C5—C6	1.384 (8)	C15—H15A	0.9600
C5—H5	0.9300	C15—H15B	0.9600
C6—H6	0.9300	C15—H15C	0.9600
C7—N1	1.292 (7)	C16—N2	1.449 (7)
C7—C8	1.446 (8)	C16—C17	1.512 (9)
C7—H7	0.9300	C16—H16A	0.9700
C8—C9	1.404 (8)	C16—H16B	0.9700
C8—C13	1.409 (8)	C17—H17A	0.9600
C9—C10	1.354 (8)	C17—H17B	0.9600
C9—H9	0.9300	C17—H17C	0.9600
C10—C11	1.430 (9)	O1—H1	0.8200
C10—H10	0.9300		
			
C2—C1—C6	118.8 (5)	C13—C12—H12	118.7
C2—C1—N1	116.7 (5)	C11—C12—H12	118.7
C6—C1—N1	124.3 (5)	O1—C13—C12	118.7 (5)
C3—C2—C1	120.1 (5)	O1—C13—C8	121.0 (6)
C3—C2—H2	119.9	C12—C13—C8	120.2 (5)
C1—C2—H2	119.9	N2—C14—C15	114.7 (6)
C2—C3—C4	122.0 (5)	N2—C14—H14A	108.6
C2—C3—I1	118.2 (4)	C15—C14—H14A	108.6
C4—C3—I1	119.8 (4)	N2—C14—H14B	108.6
C5—C4—C3	117.6 (5)	C15—C14—H14B	108.6
C5—C4—H4	121.2	H14A—C14—H14B	107.6
C3—C4—H4	121.2	C14—C15—H15A	109.5
C4—C5—C6	121.9 (6)	C14—C15—H15B	109.5
C4—C5—H5	119.0	H15A—C15—H15B	109.5
C6—C5—H5	119.0	C14—C15—H15C	109.5
C5—C6—C1	119.5 (6)	H15A—C15—H15C	109.5
C5—C6—H6	120.3	H15B—C15—H15C	109.5
C1—C6—H6	120.3	N2—C16—C17	114.0 (5)
N1—C7—C8	120.3 (6)	N2—C16—H16A	108.8
N1—C7—H7	119.8	C17—C16—H16A	108.8
C8—C7—H7	119.8	N2—C16—H16B	108.8
C9—C8—C13	117.3 (6)	C17—C16—H16B	108.8
C9—C8—C7	120.6 (5)	H16A—C16—H16B	107.6
C13—C8—C7	122.0 (6)	C16—C17—H17A	109.5
C10—C9—C8	122.5 (6)	C16—C17—H17B	109.5
C10—C9—H9	118.8	H17A—C17—H17B	109.5
C8—C9—H9	118.8	C16—C17—H17C	109.5
C9—C10—C11	120.6 (5)	H17A—C17—H17C	109.5
C9—C10—H10	119.7	H17B—C17—H17C	109.5
C11—C10—H10	119.7	C7—N1—C1	121.0 (5)
N2—C11—C12	122.1 (5)	C11—N2—C16	121.0 (5)
N2—C11—C10	121.2 (4)	C11—N2—C14	120.2 (5)
C12—C11—C10	116.7 (5)	C16—N2—C14	118.6 (5)
C13—C12—C11	122.6 (5)	C13—O1—H1	109.5
			
C6—C1—C2—C3	1.1 (8)	C10—C11—C12—C13	0.2 (8)
N1—C1—C2—C3	176.8 (5)	C11—C12—C13—O1	179.6 (5)
C1—C2—C3—C4	−1.7 (8)	C11—C12—C13—C8	−1.9 (9)
C1—C2—C3—I1	−179.9 (4)	C9—C8—C13—O1	−178.9 (5)
C2—C3—C4—C5	1.5 (9)	C7—C8—C13—O1	2.5 (8)
I1—C3—C4—C5	179.7 (4)	C9—C8—C13—C12	2.7 (8)
C3—C4—C5—C6	−0.8 (9)	C7—C8—C13—C12	−175.9 (5)
C4—C5—C6—C1	0.3 (9)	C8—C7—N1—C1	172.9 (5)
C2—C1—C6—C5	−0.4 (8)	C2—C1—N1—C7	152.9 (5)
N1—C1—C6—C5	−175.8 (5)	C6—C1—N1—C7	−31.6 (8)
N1—C7—C8—C9	−179.0 (6)	C12—C11—N2—C16	177.3 (5)
N1—C7—C8—C13	−0.4 (9)	C10—C11—N2—C16	−2.5 (8)
C13—C8—C9—C10	−1.9 (9)	C12—C11—N2—C14	−8.6 (8)
C7—C8—C9—C10	176.7 (6)	C10—C11—N2—C14	171.6 (5)
C8—C9—C10—C11	0.3 (9)	C17—C16—N2—C11	85.1 (7)
C9—C10—C11—N2	−179.6 (5)	C17—C16—N2—C14	−89.0 (7)
C9—C10—C11—C12	0.6 (7)	C15—C14—N2—C11	−76.5 (8)
N2—C11—C12—C13	−179.5 (5)	C15—C14—N2—C16	97.7 (7)

### Hydrogen-bond geometry (Å, º)

**Table:**

*D*—H···*A*	*D*—H	H···*A*	*D*···*A*	*D*—H···*A*
O1—H1···N1	0.82	1.85	2.577 (6)	147
